# Peripheral Leukocytosis Is Inversely Correlated with Intratumoral CD8+ T-Cell Infiltration and Associated with Worse Outcome after Chemoradiotherapy in Anal Cancer

**DOI:** 10.3389/fimmu.2017.01225

**Published:** 2017-09-29

**Authors:** Daniel Martin, Franz Rödel, Ria Winkelmann, Panagiotis Balermpas, Claus Rödel, Emmanouil Fokas

**Affiliations:** ^1^Department of Radiotherapy and Oncology, Goethe University Frankfurt, Frankfurt, Germany; ^2^German Cancer Research Center (DKFZ), Heidelberg, Germany; ^3^German Cancer Consortium (DKTK), Partner Site Frankfurt am Main, Frankfurt, Germany; ^4^Senckenberg Institute for Pathology, Goethe University Frankfurt, Frankfurt, Germany

**Keywords:** anal cancer, leukocytosis, immune microenvironment, tumor-infiltrating-lymphocytes, neutrophils, CD8, myeloperoxidase

## Abstract

Peripheral blood leukocytosis has been implicated in promoting tumor progression leading to worse survival, but the mechanisms behind this phenomenon remain unexplored. Here, we examined the prognostic role of pretreatment white blood cell (WBC) count and clinicopathologic parameters in the context of CD8+ tumor-infiltrating lymphocytes (TIL) and myeloperoxidase+ tumor-associated neutrophils (TANs) in patients with anal squamous cell carcinoma (ASCC) treated with definitive chemoradiotherapy (CRT). After a median follow-up of 26 months, leukocytosis correlated with advanced T-stage (*p* < 0.001) and N-stage (*p* < 0.001), and predicted for worse distant-metastasis-free survival (*p* = 0.006), disease-free-survival (DFS, *p* = 0.029), and overall survival (*p* = 0.013). Importantly, leukocytosis was associated with a lower intraepithelial CD8+ TIL density (*p* = 0.014), whereas low CD8+ TIL expression in the intraepithelial compartment was associated with worse DFS (*p* = 0.028). Additionally, high TAN expression in the peritumoral compartment was associated with a significantly lower density of CD8+ TIL (*p* = 0.039), albeit, TAN expression lacked prognostic value. In conclusion, leukocytosis constitutes an important prognostic marker in ASCC patients treated with CRT. In conjunction with intratumoral TIL and TAN, these data provide for the first time important insight on the correlation of peripheral blood leukocytosis with the intratumoral immune contexture and could be relevant for future patient stratification using immunotherapies in ASCC.

## Introduction

Anal squamous cell carcinoma (ASCC) arises from the anal canal and is a rather rare malignancy with an incidence of two to three in 100,000 per year in the general population (1). While organ-preserving definitive chemoradiotherapy (CRT) remains standard of care, locoregional persistence or relapse still occurs in up to 30% of all patients (2, 3). Large randomized trials have already established T-stage, N-stage, and gender as prognostic factors (4, 5). Nevertheless, due to the heterogeneity in treatment outcomes, there is still a need for reliable biomarkers to further stratify patients to treatment escalation or de-escalation.

The development of ASCC is associated with infection with high-risk strains of human papilloma virus (HPV) in 70–90% of cases (6, 7). HPV positivity, as measured by immunohistochemistry or polymerase chain reaction, is associated with better treatment outcome after (chemo)radiotherapy (7, 8). Furthermore, there seems to be a link between HPV and immune contexture, as HPV positivity is associated with a more favorable tumor microenvironment (TME) (9).

The role of the immune TME in mediating cancer development and progression gained attraction in recent years. The positive prognostic impact of tumor-infiltrating lymphocytes (TILs) has been reported in several malignancies (10), including ASCC (9, 11–13). CD8+ T-cells have cytotoxic capabilities due to granules secreting perforin and granzyme B, which are released upon detection of antigens *via* the major histocompatibility complex I and T-cell receptor (TCR) (14). In contrast, myeloid cells like neutrophils remain unexplored in ASCC. Neutrophils aid tumor progression and resistance to conventional anticancer treatments by mediating angiogenesis and suppression of CD8+ TIL-mediated antitumor immunity *via* secretion of inhibitory factors, e.g., inducible nitric oxide synthase (iNOS), arginase 1 (ARG1), and transforming-growth-factor beta (TGFβ) (15–17).

The negative impact of elevated pretreatment white blood cell (WBC) count in blood samples has already been reported in cervical and colorectal cancer (18–20). In ASCC, the same effect was described in recent retrospective studies (21–23). Leukocytosis was also correlated with adverse cancer-specific survival and overall survival (OS) in the ACT-I clinical trial (24). Leukocytosis in solid tumors can be caused directly by tumor cells *via* secretion of stimulatory factors such as granulocyte-colony-stimulating factor (G-CSF), granulocyte-macrophage-colony-stimulating factor, and upregulation of C-X-C chemokine receptor 2 (CXCR2) ligands (17). Nevertheless, no explanation for this observation has been provided so far.

Platelets play an important role in cancer progression. Clinical and preclinical evidence suggests that platelets can help tumor cells resist apoptosis, induce angiogenesis, facilitate chemoresistance, and help immune evasion during generation of metastasis (25, 26). Elevated pretreatment platelets are associated with worse outcome in colorectal cancer patients (27) and a higher propensity for metastasis (28).

In this study, we provide insight on the worse clinical outcome in patients with leukocytosis in correlation CD8+ TILs and tumor-associated neutrophils (TAN) in ASCC.

## Materials and Methods

### Patients and Treatment Protocol

We identified 79 patients with newly diagnosed ASCC with available pretreatment formalin-fixed paraffin embedded (FFPE) tissues at our department. All patients were treated between 03/2003 and 05/2016 with definitive CRT and were routinely subjected to physical and rectal-digital examination, proctoscopy with biopsy, CT/MRI of the abdomen and pelvis, chest X-ray, serum chemistry, and complete blood count before initiation of therapy. Patients were staged according to UICC Version 8. Analysis was done following an institutional review board approval.

Chemotherapy was applied in the first and fifth week of radiotherapy (RT) and consisted of 5-fluorouracil (1,000 or 800 mg/m^2^/day) as 4- or 5-day continuous infusion and Mitomycin C given as an intravenous bolus (10 mg/m^2^) on day one of each cycle. RT was applied using either 3D-conformal RT or intensity-modulated-RT (IMRT). Patients were treated with median total dose of 59.4 Gy (range, 34.2–64.8) with daily fractions of 1.8 or 2 Gy. 69 patients were treated with a median external boost dose of 9 Gy (range, 3.6–19.8).

### Follow-up

Initial assessment of patients was done 8–10 weeks after completion of therapy and, afterward, every 3 months for the first 2 years followed by 6-month intervals. Examination on follow-up consisted of digital rectal examination, proctoscopy (with biopsies in case of suspicious lesions), and pelvic CT/MRI scan. No detectable tumor at first follow-up was defined as complete response (CR).

### Immunohistochemistry

Immunohistochemical staining of myeloperoxidase (MPO) and CD8 was performed by a horseradish-peroxidase technique using a DAKO Autostainer Link 48 (DAKO, Hamburg, Germany) with standardized Dako EnVision™ FLEX Blocking reagent (K800, DAKO) and polyclonal MPO antibody MPO (dilution 1:1,000; A0398, DAKO) and CD8 (1:100, clone C8/144B; DAKO M7103). Next, dextran polymer conjugated horseradish-peroxidase and 3,3′-diaminobenzidine chromogen was used for visualization and hematoxylin solution (Gill 3, Sigma-Aldrich, Munich, Germany) for counterstaining. Negative control slides in the absence of primary antibodies were included for each staining.

The scoring of expression of MPO and CD8+ TIL was done semiquantitatively as described before (9). Categories for scoring were: (1) no, or sporadic cells; (2) moderate number of cells; (3) abundant occurrence of cells; (4) highly abundant occurrence of cells was done for both, intraepithelial, and stromal compartments (×10 magnification) separately, and a total score was generated by addition of both scores as previously described (29, 30). We used the median value as cutoff for both compartments and the total score to divide the patient cohort in patients either having a low or high score. Images were acquired with the AxioImager Z1 microscope using the Axiovision 4.6 software (Zeiss, Germany). To minimize interobserver variability, two investigators (Daniel Martin and Emmanouil Fokas) without knowledge of the clinicopathologic data performed scoring. In cases of discrepancy, a final decision was made after additional examination of the specimens.

### Peripheral Blood Counts

We derived the following baseline WBC counts and platelet counts from our hospital database. Differential blood counts were not available for a large proportion of patients. Baseline was defined as either the morning of treatment initiation or up to 4 days before this date. Leukocytosis was defined as having a WBC count equal or above the median. Additional analysis was done using a traditional cutoff of 10/nl.

### Statistical Analysis

Differences between groups were assessed using Pearson’s Chi-squared test for categorical variables and using the non-parametric Kruskal–Wallis test for continuous variables. Correlations were assessed using the Spearman’s rank correlation coefficient. Survival times were calculated from start of CRT to the dates of respective events or last follow-up. Cumulative incidence of local failure was assessed using non-complete response at first restaging or locoregional recurrence after initial complete response as event. Disease-free survival (DFS) was calculated using the date of diagnosis of locoregional failure, distant metastases, or death of any cause. OS was calculated with death of any cause as the respective event. Remaining patients were censored for the respective end point. Survival curves were plotted using Kaplan–Meier plots and differences between curves were analyzed using the log-rank test. The Cox proportional hazard model was used for univariate analysis in order to assess the influence of WBC count and platelet count as a continuous variable. The assumption of proportional hazard was tested by assessing the scaled Schoenfeld residuals. Due to the low number of events, we were not able to conduct a multivariate analysis without risking overfitting (31). Statistical analysis was performed using R (Version 3.3) (32). A *p*-value of <0.05 was considered significant.

## Results

### Patient and Treatment Characteristics

**Figure 1:**
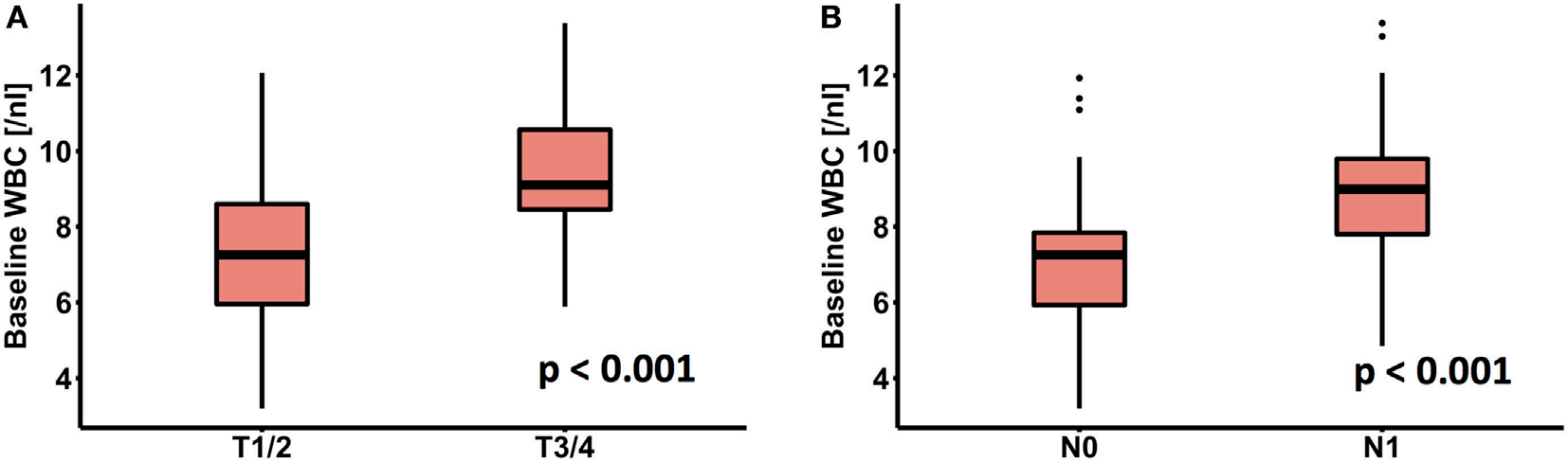
Association of peripheral white blood cell (WBC) with **(A)** T-stage and **(B)** N-stage.

Patient and treatment characteristics are summarized in Table 1. Advanced T-stage (*p* < 0.001) and N-stage (*p* < 0.001) were significantly associated with an elevated WBC count (Figures 1A,B). There was a significant positive correlation between WBC count and platelet count (*r* = 0.59, *p* < 0.001), whereas there was no significant difference according to gender, grading, and age.

**Table 1:** Patients, tumor, and blood characteristics.

		Median (range) or *n* (%)
Age, years		58 (36–84)
Sex	Male	34 (43)
	Female	45 (57)
T-stage	T1	22 (28)
	T2	35 (44)
	T3	16 (20)
	T4	6 (8)
N-stage	N0	49 (62)
	N1	30 (38)
Grading	G1	4 (5)
	G2	50 (63)
	G3	21 (27)
	Gx	4 (5)
**Pretreatment blood counts**		
Leukocytes (/nl)		7.69 (3.2–13.38)
Platelets (/nl)		266 (111–531)
Hemoglobin (mg/dl)		13.5 (10.2–16.2)
**Radiotherapy (RT)**		
RT modality	3D conformal RT	32
	Intensity-modulated-RT	47
Total dose (Gy)		59.4 (34.2–64.8)
Boost dose (Gy)		9 (0–19.8)

### Leukocytosis and Intratumoral Immune Contexture

**Figure 2:**
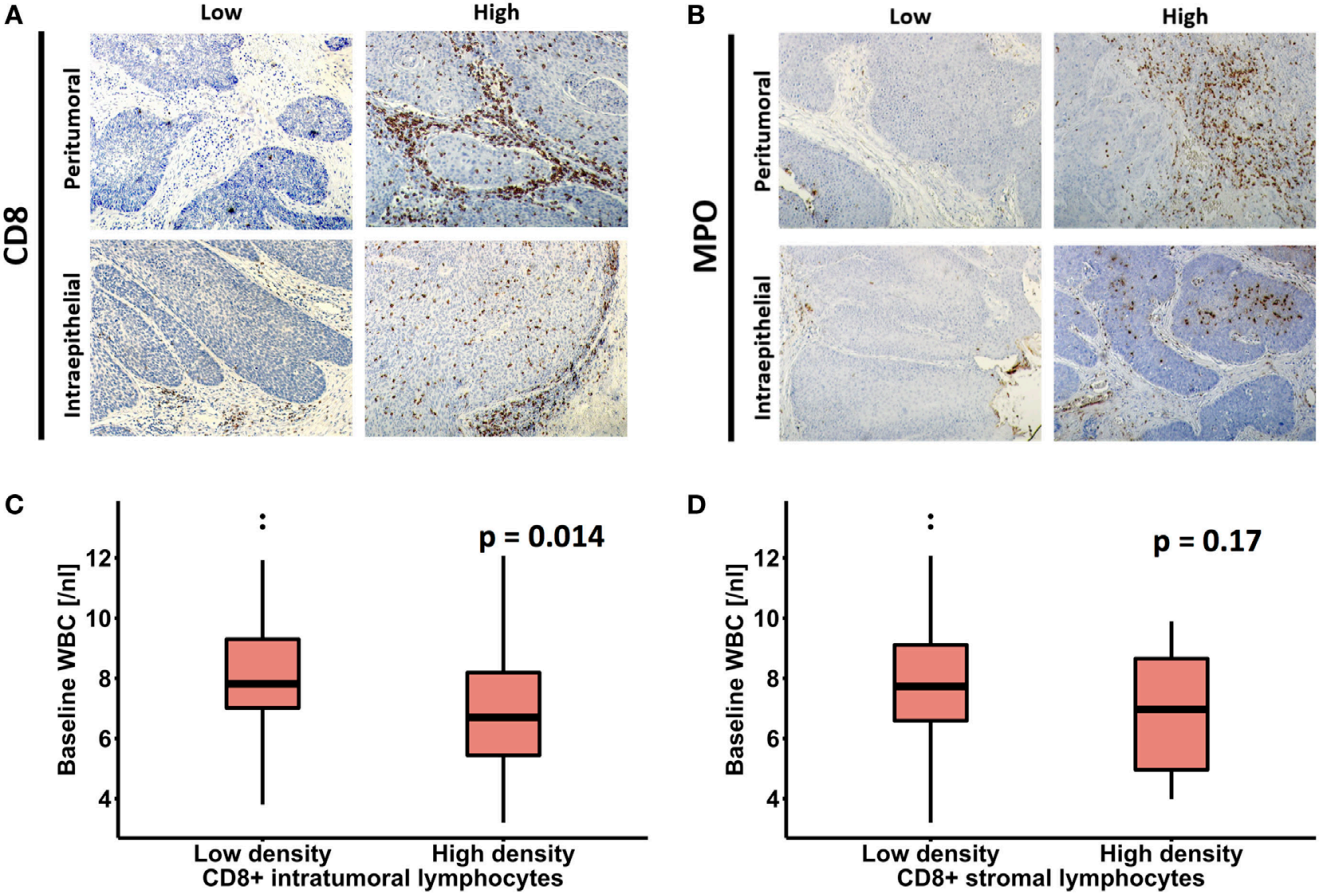
**(A,B)** Example of immunohistochemical images showing high and low CD8 and myeloperoxidase (MPO) expression in pretreatment biopsies of patients with anal squamous cell carcinoma. Association of peripheral white blood cell (WBC) count with **(C)** intratumoral and **(D)** peritumoral (stromal) CD8+ T-cells.

The results of CD8 and MPO staining in pretreatment biopsies and correlations with clinicopathologic parameters are summarized in Table 2 and Table S1 in Supplementary Material. Male patients had a significantly higher intratumoral MPO+ TAN density compared to females (*p* = 0.03). There was no association between tumor size, nodal status, age, CD8+ TIL, or MPO+ TAN densities regardless of compartment. High TAN expression was associated with a significantly lower density of CD8+ TIL in the peritumoral (stromal) areas (*p* = 0.029). The same significant association also existed between peritumoral MPO+ TAN and CD8+ peritumoral TIL (*p* = 0.039). Representative images of low and high intratumoral and peritumoral CD8+ TIL and MPO+ TAN expression are shown in Figures 2A,B.

**Table 2:** Correlation of clinicopathological parameters with MPO and CD8 score according to different tumor compartments.

Parameter	MPO stroma			MPO tumor			MPO total			CD8 stroma			CD8 tumor			CD8 total		
	Low, *n* (%)	High, *n* (%)	*p*-Value	Low, *n* (%)	High, *n* (%)	*p*-Value	Low, *n* (%)	High, *n* (%)	*p*-Value	Low, *n* (%)	High, *n* (%)	*p*-Value	Low, *n* (%)	High, *n* (%)	*p*-Value	Low, *n* (%)	High, *n* (%)	*p*-Value
**T-stage**																		
T1/2	17 (68)	40 (74)	0.57	47 (69)	10 (91)	0.13	17 (71)	40 (73)	0.86	48 (70)	9 (90)	0.18	36 (67)	21 (84)	0.11	29 (66)	28 (80)	0.17
T3/4	8 (32)	14 (26)		21 (31)	1 (9)		7 (29)	15 (27)		21 (30)	1 (10)		18 (33)	4 (16)		15 (34)	7 (20)	
**N-stage**																		
N0	15 (60)	34 (63)	0.8	42 (62)	7 (64)	0.9	14 (58)	35 (64)	0.65	41 (59)	8 (80)	0.21	30 (56)	19 (76)	0.08	24 (55)	25 (71)	0.12
N1	10 (40)	20 (37)		26 (38)	4 (36)		10 (42)	20 (36)		28 (41)	2 (20)		24 (44)	6 (24)		20 (45)	10 (29)	
**G**																		
G1/2	15 (60)	38 (75)	0.2	44 (67)	9 (90)	0.13	14 (58)	39 (75)	0.14	46 (70)	7 (70)	0.98	33 (65)	20 (80)	0.17	26 (63)	27 (77)	0.19
G3	10 (40)	13 (25)		22 (33)	1 (10)		10 (42)	13 (25)		20 (30)	3 (30)		18 (35)	5 (20)		15 (37)	8 (23)	
**Gender**																		
Male	10 (40)	24 (44)	0.7	26 (38)	8 (73)	**0.03**	9 (38)	25 (45)	0.51	32 (46)	2 (20)	0.12	23 (43)	11 (44)	0.91	20 (45)	14 (40)	0.63
Female	15 (60)	30 (56)		42 (62)	3 (27)		15 (62)	30 (55)		37 (54)	8 (8)		31 (57)	14 (56)		24 (55)	21 (60)	
**Age (years)**																		
<58	13 (52)	25 (46)	0.64	30 (44)	8 (73)	0.08	12 (50)	26 (47)	0.82	34 (49)	4 (40)	0.58	26 (48)	12 (48)	0.99	23 (52)	15 (43)	0.4
≥58	12 (48)	29 (54)		38 (56)	3 (7)		12 (50)	29 (53)		35 (51)	6 (60)		28 (52)	13 (52)		21 (48)	20 (57)	
**CD8 stroma**																		
Low	19 (76)	50 (93)	**0.039**	58 (85)	11 (100)	0.18	18 (75)	51 (93)	**0.029**			–			–			–
High	6 (24)	4 (7)		10 (15)	0 (0)		6 (25)	4 (7)										
**CD8 tumor**																		
Low	16 (64)	38 (70)	0.57	46 (68)	8 (73)	0.74	15 (63)	39 (71)	0.46			–			–			–
High	9 (36)	16 (30)		22 (32)	3 (27)		9 (37)	16 (29)										
**CD8 total**																		
Low	12 (48)	32 (59)	0.3.5	37 (54)	7 (64)	0.57	11 (46)	33 (60)	0.24			–			–			–
High	13 (52)	22 (41)		31 (46)	4 (36)		13 (54)	22 (40)										

*MPO, myeloperoxidase*.

*Significant values have been marked with bold*.

Regarding the correlation of peripheral WBC and TME, pretreatment tumor specimens with a high intraepithelial CD8+ TIL expression had a significantly lower baseline WBC count (*p* = 0.014) compared to patients with no or only low infiltration, whereas there was no significant correlation between WBC and peritumoral (stromal) CD8+ TIL (*p* = 0.17) (Figures 2C,D). Similarly, we failed to detect a significant association between tumor MPO expression and peripheral WBC. Of note, patients with a high intraepithelial infiltration with CD8+ TIL had lower baseline platelets (Figure S1 in Supplementary Material).

### Treatment Outcome

The median follow-up time was 26 months (range, 0–158). One patient died during therapy and 62 (78%) patients had a complete response (CR) after CRT. Locoregional failure after initial CR occurred in 10 (16%) patients, whereas 7 (9%) patients were diagnosed with distant metastases. Furthermore, 12 (15%) patients died during follow-up; 6 (8%) due to ASCC, 2 (3%) due to treatment-related complications, and 4 (5%) due to unrelated disease.

**Figure 3:**
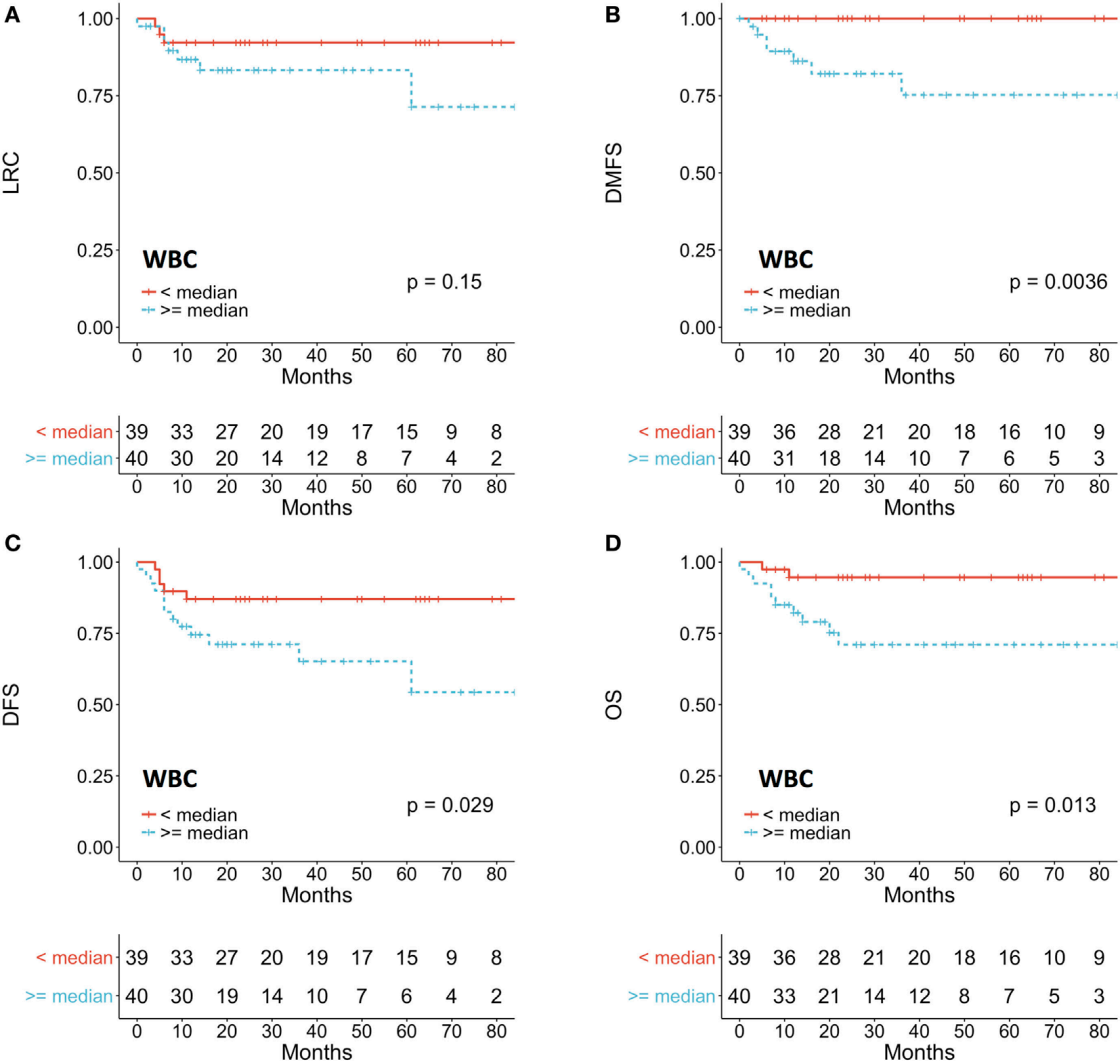
Prognostic impact of white blood cell (WBC) count on **(A)** LRC, **(B)** DMFS, **(C)** DFS, **(D)** OS. Patients were dichotomized according to the median value of WBC count. LRC, locoregional control; DMFS, distant-metastasis-free survival; DFS, disease-free survival; OS, overall survival.

Patients with a pretreatment leukocytosis (≥median WBC count) had a significantly worse distant-metastasis-free survival (DMFS) (*p* = 0.0063), DFS (*p* = 0.029), and OS (*p* = 0.013) (Figures 3A–D). In an additional analysis that used WBC ≥10/nl as a cutoff as previously described (21, 23), leukocytosis was seen in 10 (13%) patients and was associated with a significantly worse local control (*p* = 0.048), DMFS (*p* = 0.0021), DFS (*p* = 0.011), and OS (*p* = 0.0067) (Figure S2 in Supplementary Material). To evaluate WBC count as a continuous variable, we used a cox proportional hazard model. Increase of WBC count was significantly associated with worse DMFS (HR 1.76; CI 1.22–2.55; *p* = 0.003), DFS (HR 1.31; CI 1.06–1.62; *p* = 0.014), and OS (HR 1.43; CI 1.09–1.86; *p* = 0.009).

Regarding the impact of pretreatment platelet count, we found no significant prognostic impact using several cutoffs for dichotomization. In an additional univariate cox regression analysis using platelet count as a continuous variable, however, elevated platelet count was associated with worse DMFS (HR 1.009, 95% CI 1–1.018, *p* = 0.048).

**Figure 4:**
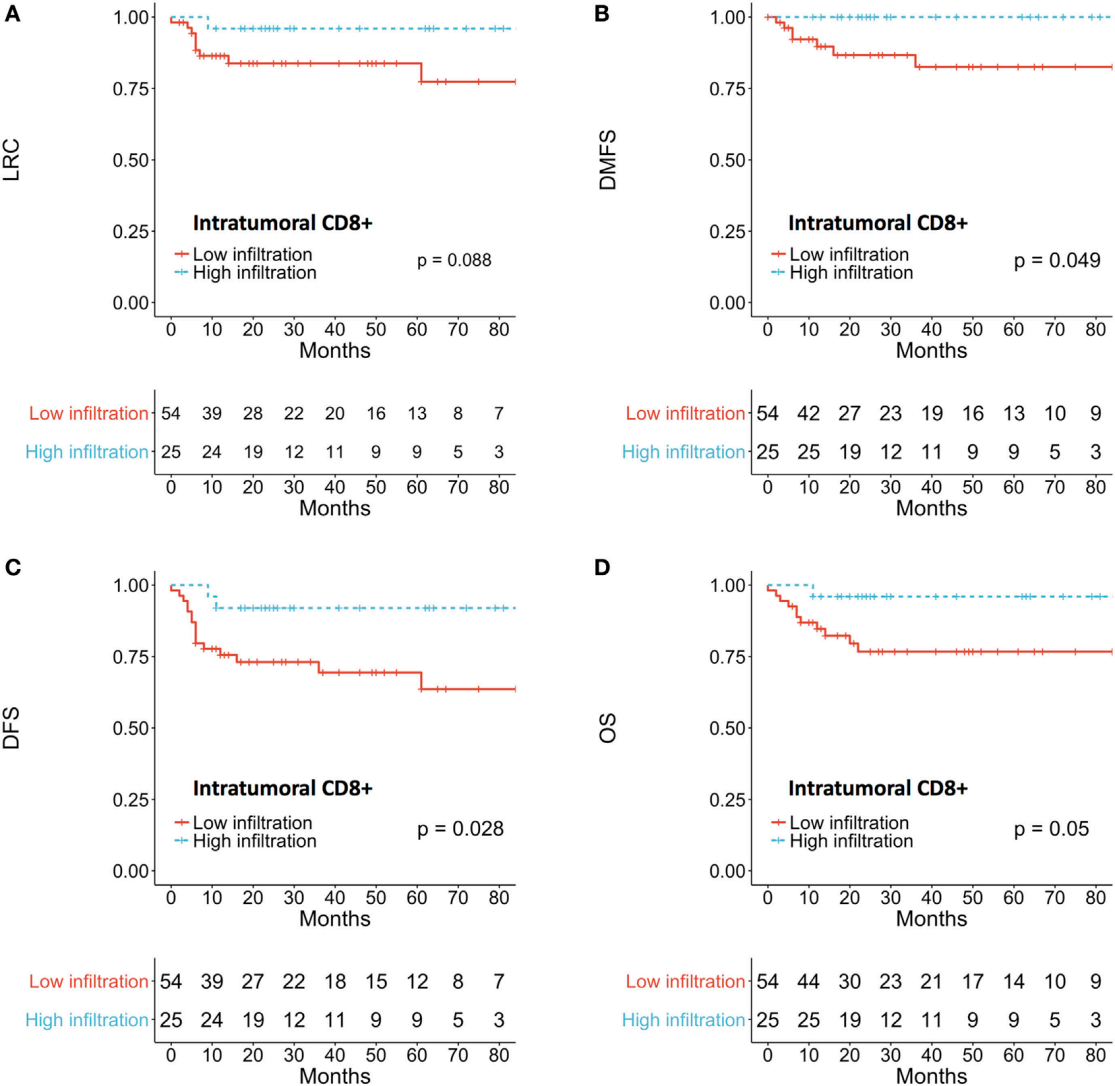
Prognostic impact of intratumoral CD8+ T-cells on **(A)** LRC, **(B)** DMFS, **(C)** DFS, **(D)** OS. Patients were dichotomized according to the median intratumoral score. LRC, locoregional control; DMFS, distant-metastasis-free survival; DFS, disease-free survival; OS, overall survival.

A higher density of intratumoral CD8+ TIL was associated with a better DMFS (*p* = 0.049) and DFS (*p* = 0.028) (Figures 4A–D). There was no impact of the total score or the peritumoral density of CD8+ TIL on treatment outcome. Similarly, MPO+ neutrophil expression did not affect prognosis in our series.

## Discussion

In the present study, we aimed to investigate the clinical impact of peripheral blood leukocytosis in the context of intratumoral immune profile. Patients with ASCC that have baseline leukocytosis presented a worse outcome after CRT in line to previous observation in ASCC (21, 23) and cervical cancer (18, 20). Our data confirm these results using the established cutoff point of 10/nl, but we found worse DFS and DMFS also in patients with moderately elevated WBC, based on median cutoff value.

In our work, patients with leukocytosis had a significantly lower intratumoral CD8+ TIL expression and vice versa. Leukocytes are important in acute inflammation to eliminate pathogens but, in a chronically activated state, they can promote tumor progression by secretion of TGFβ, epidermal-growth-factor, and proteolytic enzymes (33). Importantly, leukocytosis as part of chronic inflammation can lead to inhibition of CD8+ T-cells *via* upregulation of programmed death 1 on T-cells and myeloid cells (34) that is in line to our findings. To the best of our knowledge, this phenomenon has not been reported yet and it proposes an additional explanation for the adverse role of leukocytosis in malignancies.

The role of TAN in ASCC has not been previously investigated. Notably, we found an inverse correlation between peritumoral TAN and CD8+ TIL densities. Several mechanisms have been implicated in the immunosuppressive effect of TAN. First, secretion of ARG1 lowers the expression of the ζCD3 chain of the TCR leading to reduced activity (35, 36). Second, nitration of CC-chemokine ligand 2 by neutrophil-derived reactive nitrogen species reduces T-cell infiltration (37). Third, iNOS released by neutrophils inhibits proliferation of CD8 lymphocytes (16). Fourth, TGFβ1 signaling downregulates interferon gamma (IFNγ) in CD8+ T-cells (38). The positive prognostic impact of high CD8+ TIL density has already been demonstrated in ASCC (9, 11–13).

Previous studies have reported strong association of leukocytosis with peripheral blood neutrophilia in ASCC (23) and cervical cancer (18, 20, 39). The impact of leukocytosis on DFS and OS in our study was mainly due to distant metastases, and experimental models showed that neutrophils play a major role in early metastatic spread (40). Tumors promote neutrophil release *via* G-CSF and CXCR2 ligands (41, 42) that can lead to chemo- and radioresistance (39, 43). Of note, we failed to identify a significant correlation between total peripheral WBC and intratumoral neutrophils, which could be attributed to the fact that WBC count incorporates various peripheral blood cell populations.

The negative impact of elevated platelets on DMFS is in line with preclinical evidence that suggests an important role for platelets in metastasis (26). Similar data have been reported before in other tumor types, such as colorectal cancer (27, 28). Also, prophylactic aspirin that inhibits platelet activation has been shown to decrease metastatic incidence and cancer mortality (44).

We did not observe any significant prognostic influence of TAN on treatment outcome. Of note, a high variance has been reported regarding the prognostic value of neutrophils (45–50). One possible explanation for these discrepancies in literature could be the heterogeneous treatments and cohorts, and the use of different markers to evaluate TAN (CD11b, CD15, MPO). Another explanation could be different compositions of the TME between malignancies. In cervical cancer, a negative prognostic impact of TAN is reported (51), whereas in squamous cell carcinoma of the head and neck, studies have reported positive and negative prognostic value (52, 53). Several approaches for targeting TAN are under clinical evaluation, for example, by blocking CXCR2 using the inhibitor reparixin (54). Interestingly, preclinical evidence has also suggested a synergistic effect of combining CXCR2 blockade with immune checkpoint inhibition (55).

Our study has several limitations. First, differential baseline blood count values, including neutrophils and lymphocytes, were unavailable in a large proportion of patients. Second, the retrospective design could have resulted in selection bias. Third, although our median follow-up of 26 months was relatively long, randomized trials have been reported with longer follow-up time. Fourth, immunohistochemical staining scoring was performed using a non-automated system due to the lack of international consensus and pathology standardization that constitutes another limitation of our work. These results clearly need to be validated in other cohorts, preferably in a prospective manner. In that context, we and others have previously investigated additional potential biomarkers relevant to the immune microenvironment, such as EGFR, surviving, Plk3, FOXP3, and PD-1/PD-L1 (9, 56–60). These could be potentially exploited in the future to generate robust prognostic models with several biomarkers to guide treatment stratification for future trials.

In conclusion, we here demonstrate that peripheral leukocytosis was associated with adverse clinical outcome in patients with ASCC treated with definitive CRT. This is the first study to show an inverse correlation between peripheral blood leukocytosis and intratumoral CD8+ TIL as well as between peritumoral MPO+ TAN and stromal CD8+ TIL that provide important mechanistic insight on the adverse impact of peripheral leukocytosis and the interplay between different immune cell populations in ASCC. These findings add to the previous literature on the role of leukocytosis and immune contexture in mediating response to treatment in ASCC and should lead to further investigations into the immune microenvironment of this disease.

## Ethics Statement

This study was carried out in accordance with the recommendations of the ethics committee of the University Hospital Frankfurt am Main and approval from the institutional review board. All patients gave written informed consent in accordance with the Declaration of Helsinki.

## Author Contributions

All authors designed and drafted the project. Clinical data collection and scoring of histochemical staining was done by DM, FR, PB, CR, and EF; RW performed pathological evaluation. All authors contributed to statistical analyses and DM, PB, and EF drafted the final manuscript with support from all co-authors. All authors performed final evaluation and agreed to be accountable for the content of the study.

## Conflict of Interest Statement

The authors declare that the research was conducted in the absence of any commercial or financial relationships that could be construed as a potential conflict of interest.

## Supplementary Material

Figure S1 Association of pretreatment platelet count with **(A)** intratumoral and **(B)** stromal CD8+ T-cells.

Figure S2 Prognostic impact of WBC on **(A)** LRC, **(B)** DMFS, **(C)** DFS, **(D)** OS. Patients were dichotomized according to a WBC count ≥ 10 nl^-1^. LRC, locoregional control; DMFS, distant-metastasis-free survival; DFS, disease-free survival; OS, overall survival.
