# Supplementary figures and images for: Peripheral Leukocytosis Is Inversely Correlated with Intratumoral CD8+ T-Cell Infiltration and Associated with Worse Outcome after Chemoradiotherapy in Anal Cancer
Source: Front Immunol. 2017 Sep 29;8:1225. doi: 10.3389/fimmu.2017.01225 (PMC5649213; doi:10.3389/fimmu.2017.01225)

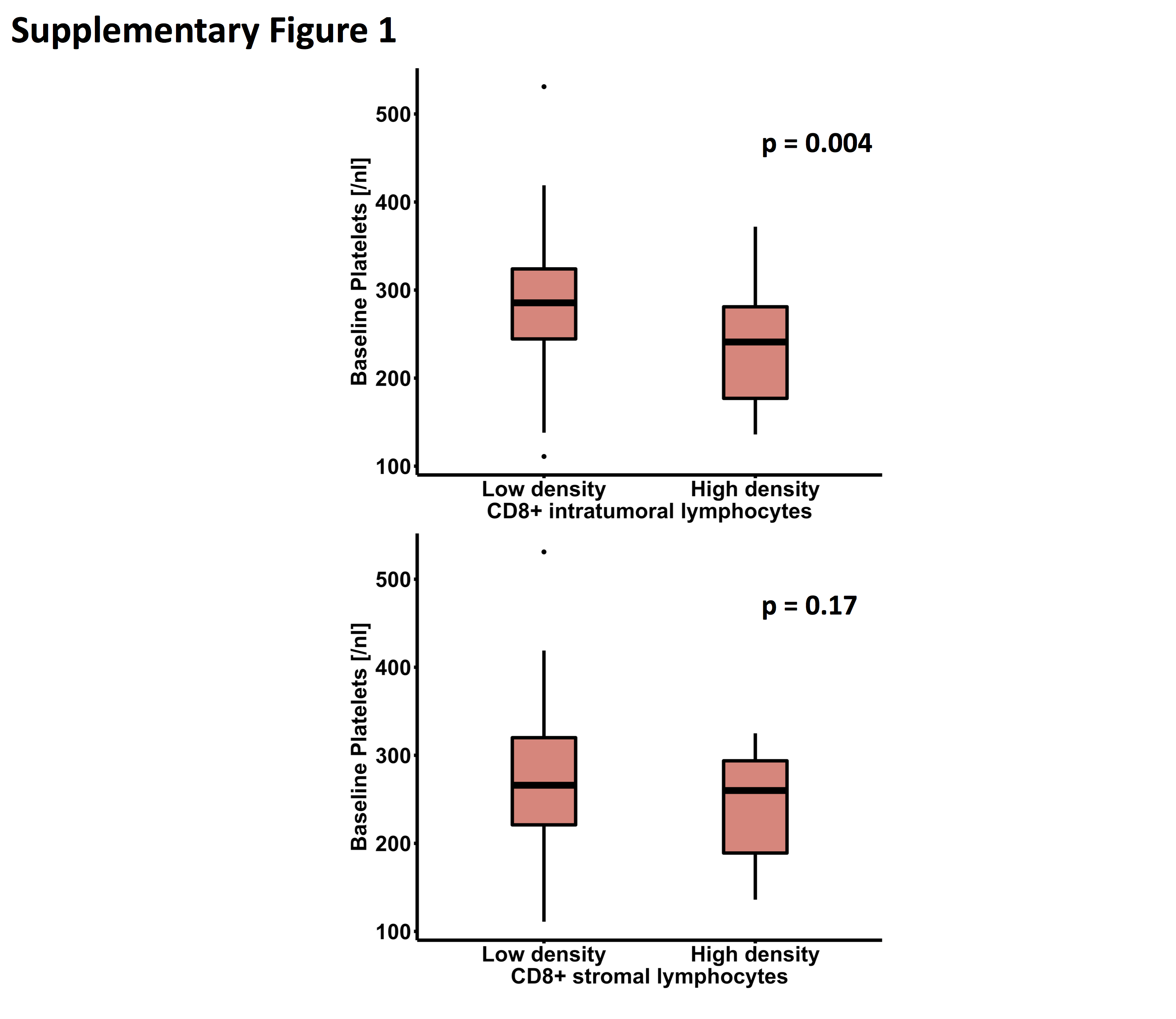

Supplement: Figure S1 — Association of pretreatment platelet count with (A) intratumoral and (B) stromal CD8+ T-cells. [file image_1.tiff]

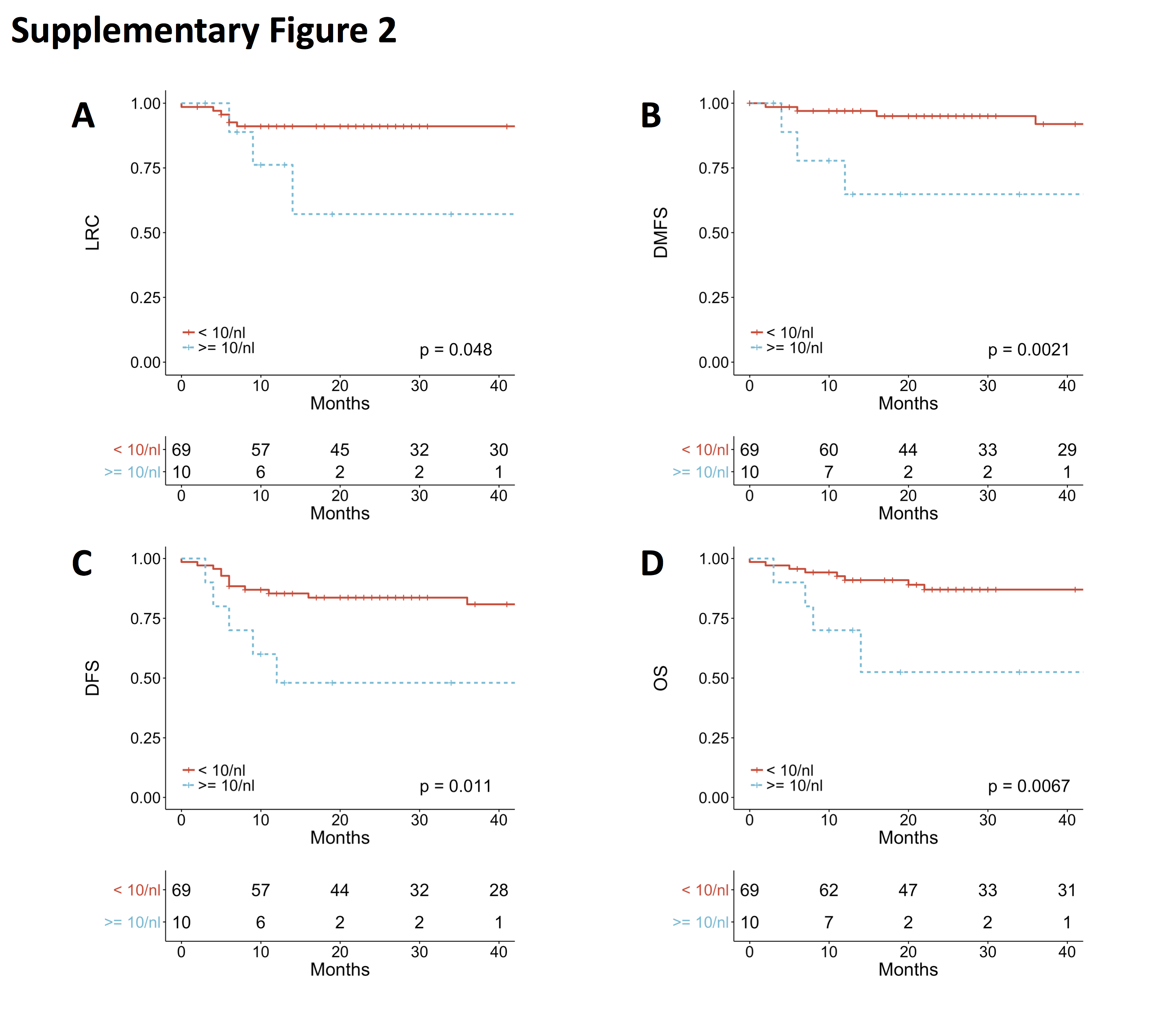

Supplement: Figure S2 — Prognostic impact of WBC on (A) LRC, (B) DMFS, (C) DFS, (D) OS. Patients were dichotomized according to a WBC count ≥ 10 nl-1. LRC, locoregional control; DMFS, distant-metastasis-free survival; DFS, disease-free survival; OS, overall survival. [file image_2.tiff]
