# Supplementary material for: Peripheral Leukocytosis Is Inversely Correlated with Intratumoral CD8+ T-Cell Infiltration and Associated with Worse Outcome after Chemoradiotherapy in Anal Cancer
Source: Front Immunol. 2017 Sep 29;8:1225. doi: 10.3389/fimmu.2017.01225 (PMC5649213; doi:10.3389/fimmu.2017.01225)
Supplement: Supplementary file 3 [file data_sheet_1.docx]

**Supplementary Table 1**. MPO scores according to different compartments

|  | Compartment | |  |
| --- | --- | --- | --- |
| MPO | **Intraepithelial, n (%)** | **Peritumoral, n (%)** | **Total, n (%)** |
| Low score | 68 (86) | 25 (32) | 24 (30) |
| High score | 11 (14) | 54 (68) | 55 (70) |
| CD8 |  |  |  |
| Low score | 54 (68) | 69 (87) | 44 (56) |
| High score | 25 (32) | 10 (13) | 35 (44) |

Variables were dichotomized according to the median score

**Supplementary Table 2**. Results of univariate cox regression analysis with baseline platelet count

|  | HR | 95% CI | p value |
| --- | --- | --- | --- |
| LRC | 1.006 | 0.998 – 1.014 | 0.139 |
| DMFS | 1.009 | 1 – 1.018 | **0.048** |
| DFS | 1.004 | 0.996 – 1.01 | 0.16 |
| OS | 1.006 | 0.999 – 1.013 | 0.111 |

Abbreviations: LRC, locoregional control; DMFS, distant-metastasis free survival; DFS, disease-free survival; OS, overall survival
